# Impact of Plasma Pre-Treatment on the Tribological Properties of DLC Coatings on PDMS Substrates

**DOI:** 10.3390/ma14020433

**Published:** 2021-01-17

**Authors:** Witold Kaczorowski, Hanna Świątek, Klaudia Łuczak, Marta Głuszek, Marian Cłapa

**Affiliations:** 1 Faculty of Mechanical Engineering, Institute of Material Sciences and Engineering, Lodz University of Technology, 94-924 Lodz, Poland; 200073@edu.p.lodz.pl (K.Ł.); mgluszek06@gmail.com (M.G.); marian.clapa@p.lodz.pl (M.C.); 2 Department of Solid State Physics, Faculty of Applied Physics and Mathematics, Gdansk University of Technology, 80-233 Gdansk, Poland; hanna.swiatek@pg.edu.pl

**Keywords:** diamond-like carbon, plasma, PDMS, RF PACVD, DBD, tribological properties

## Abstract

The processes of the deposition of carbon coatings on PDMS (polydimethylsiloxane) substrates using plasma techniques are widely used in a large number of studies, in applications ranging from electronic to biological. That is why the potential improvement of their functional properties, including tribological properties, seems very interesting. This paper presents an analysis of the impact of plasma pre-treatment on the properties of the produced diamond-like carbon (DLC) coatings, including changes in the coefficients of friction and wear rates. The initial modification processes were performed using two different techniques based on low-pressure plasma (RF PACVD, radio-frequency plasma-assisted chemical vapour deposition) and dielectric barrier discharge (DBD) plasma. The effects of the above-mentioned treatments on the geometric structure of the PDMS surface and its water contact angles and stability over time were determined. The basic properties of the DLC coatings produced on unmodified substrates were compared to those of the coatings subjected to plasma pre-treatment. The most interesting effects in terms of tribological properties were achieved after the DBD process and production of DLC coatings, achieving a decrease in wear rates to 2.45 × 10^−8^ mm^3^/Nm. The tests demonstrate that the cross-linking of the polymer substrate occurs during plasma pre-treatment.

## 1. Introduction

Carbon coatings are a very well investigated material used to improve multiple properties of the surface layer. They can be used to increase hardness [1,2,3], improve wear resistance, reduce the coefficient of friction [1,2,3,4,5] and enhance the biocompatibility of the modified surface [6,7] or its resistance to colonisation by micro-organisms [7,8]. Due to their advantages, diamond-like carbon (DLC) coatings are tested in a wide range of applications in very different areas: biomedicine [5,6,7,8,9], machining [10,11], electronics [12,13] and electrochemistry [14,15]. To date, there are many known techniques for producing DLC coatings, with the dominant methods being chemical vapour deposition (CVD) and physical vapour deposition (PVD). Therefore, it is a material that is very well investigated in terms of properties, applications and production methods. However, there are certain areas of research where the technology for producing carbon coatings is still being improved. Such areas include coatings on polymer substrates, which are dedicated to applications in biomedicine [3,7,16,17,18]. In this case, a fairly significant problem is how to achieve the suitable adhesion of the coatings to the base material, which is ultimately the property that determines wear resistance [16,17,18,19]. Other important issues are the limitations caused by the need to conduct the processes at low temperatures (suitable for polymer materials) and the low values of the Young’s modulus of polymer materials [16,20,21]. The last of the indicated limitations is directly connected with the formation of wrinkles during coating production due to the different values of the Young’s modulus of the substrate and the coating [20,21,22]. The production of carbon coatings on the surface of polymer materials is usually performed using plasma techniques, e.g., the plasma-assisted chemical vapour deposition (PA CVD) method assisted with direct current (DC) [23] or radio-frequency (RF) discharge [3,7,16,17,24,25]. In addition to these technologies, the surface of polymer materials is also modified using dielectric barrier discharge (DBD) plasma [26,27,28], which produces effects similar to those achieved with low-pressure methods.

Plasma is used to modify polymer materials such as polyethylene (PE), polyurethanes, poly(ether-ether-ketone) (PEEK), rubbers and polydimethylsiloxane (PDMS) [3,7,16,19,20,21,22,26,29,30,31]. In the case of the last, the most frequent processes include etching [16,32,33], cross-linking [16,34,35] and the deposition of thin coatings (e.g., DLC, gold or SiOx) [20,21,22,36]. Unfortunately, not all of the available modifications can produce stable properties for the surface, and their ageing manifests through a change in the water contact angle of the surface and its surface free energy [37,38]. When PDMS is, for instance, subject only to plasma pre-treatment, it is also necessary to determine the time and ageing rate of the surface, which is affected by various factors, including, in particular, the storage environment and the type and parameters of the modification [37,38]. The ageing effect is not observed after the production of DLC coatings on the PDMS substrate [16,38].

There are a number of studies referring to the possibilities of controlling the properties of surfaces with carbon coatings by modifying the parameters of their production process [16,38,39]. In the case of the radio-frequency plasma-assisted chemical vapour deposition (RF PACVD) techniques, the parameter is the negative bias, which determines the phase composition of the coatings.

The functional properties of carbon coatings can also be affected by plasma pre-treatment. This method of modification may help to produce more subtle changes in properties along the cross-section of the specimen. To achieve the required results, plasma pre-treatment has to result not only in the etching of the polymer surface but also in the cross-linking of the surface layer [16]. This method of modifying the surface layer may improve the tribological properties, particularly the wear resistance of the carbon coatings produced at a later stage.

This paper presents the results of the tribological tests of DLC coatings produced using the RF PACVD technique on a PDMS substrate with three different methods: without pre-treatment and using pre-treatment in low-pressure radio-frequency plasma and in DBD plasma in atmospheric air. The paper also describes how plasma pre-treatment affects the structure of the PDMS surface.

## 2. Materials and Methods

### 2.1. PDMS Substrate Preparation

Polymer substrates made of the PDMS material with the Sylgard 184 trademark (by Dow Corning Inc., Midland, MI, USA) were prepared as recommended by the manufacturer. After a thorough mixing of the base with the curing agent at a 10:1 ratio and degassing, liquid PDMS was poured onto Petri dishes. The material prepared as described above was annealed at 80 °C for 2 h. After annealing, 25 mm discs with a thickness of 2 mm were cut out for the tests. Apart from the modification processes, the discs were also washed for 10 min in isopropyl alcohol and dried in compressed air.

### 2.2. Plasma Modification of PDMS Substrates

During the plasma pre-treatment using low-pressure radio-frequency plasma in the RF PACVD equipment (TUL, Lodz, Poland), the PDMS substrates were located on an RF electrode. The treatment was carried out under a pressure of 100 Pa and with a compressed air flow rate of 60 sccm, using a 500 V negative bias, for 60 and 180 s (the power density was approximately 300 mW/cm^2^). During the use of DBD plasma, the substrates were placed in an air gap between two electrodes, with an approx. 2 mm distance between the modified surface and the top electrode [40,41]. The process was conducted with a voltage of approx. 5 kV. The reactor power density was approximately 30 mW/cm^2^. The modifications were performed for 60 and 180 s. The substrates prepared as described above were used to produce DLC coatings.

The carbon coatings were produced using the same RF PACVD equipment [7,16,17,38]. In each case, the process was conducted with the same parameters. Methane was supplied to the working chamber with a flow rate of 60 sccm, and the negative bias was maintained at 500 V (the power density was approximately 700 mW/cm^2^). The duration of the process was 300 s. This method was used to prepare five batches of specimens with DLC coatings (Table 1). These included one specimen without plasma pre-treatment, two specimens with pre-treatment in the RF PACVD reactor and two further specimens pre-treated in DBD atmospheric plasma.

### 2.3. Characterisation Methods

The morphology and topography of the analysed surfaces were examined using a AFM Multimode microscope equipped with a Nanoscope V controller (Bruker Corporation, Billerica, MA, USA) [16,36]. All the examinations were carried out in the tapping mode, using silicon tips with a nominal resonance frequency of 325 kHz, spring constant of 40 N/m and tip radius <20 nm. The Nanoscope 7.3 software was used for the examinations, and image processing was carried out in the Mountains-Map 5 software (Version 5, Digital Surf, Besancon, France). Each specimen was scanned with two areas: 10 × 10 µm and 100 × 100 µm. The 100 × 100 µm scans were used to determine the most important roughness parameters of the examined surfaces (Ra, Rz and Rq) according to our earlier publications.

The water contact angles were analysed using the Drop Shape Analyzer—DSA25 (KRÜSS GmbH, Hamburg, Germany) [38]. This examination was based on measurements using the sessile drop technique. The test liquid was deionised water, introduced each time in a volume of 0.8 µL onto the tested surface. The modified specimens were analysed immediately after the modification (approx. 6 min after being removed from the chamber of the plasma equipment) until the 216th hour. Due to the fast changes in the water contact angles of the specimens that had been subjected to initial modification in RF PACVD and DBD plasma, five measurements were carried out for the specimens during the first day between the 6th minute and 24th hour. Only 3 measurement points were included in the same time range during the tests for stable DLC coatings. The subsequent stages of the tests were performed in the same way for each modification, with a 24 h interval between successive measurements.

The chemical structure of the specimens was analysed using the inVia Confocal Raman Microscope manufactured by Renishaw (Gloucestershire, UK) [16,38]. All the tests were carried out using a laser excitation wavelength of 532 nm and 100× objective. The measurements were conducted in both the ranges of 100–3200 and 900–2000 cm^−1^. The first of them allowed determining the chemical structure of the PDMS substrates before the plasma modifications, and the second enabled an accurate characterisation of the produced DLC coatings. The separation of the spectra into characteristic peaks was performed with the PeakFit 4.12 software (Version 4.12, Seasolve, San Jose, CA, USA).

The tests of tribological properties were carried out using the T-11 M tribotester (ITeE-PIB Radom, Radom, Poland) with the ball-on-disc configuration, under dry friction conditions [3,17]. The counterpart was an Al_2_O_3_ ceramic ball with a diameter of 8 mm. The primary parameters of the test were as follows: a sliding velocity of 0.05 m/s, 5 N load, and test duration of 4000 s. The tests were carried out at 25 °C and a humidity of approx. 25%. After the tests, the wear marks were characterised by profilometry using the Homell Tester T-1000 (JENOPTIK Industrial metrology, Villingen-Schwenningen, Germany) and by optical microscopy using the Nikon Eclipse MA200 microscope (NIKON, Tokyo, Japan).

## 3. Results and Discussion

### 3.1. Surface Geometrical Structure

**Figure 1:**
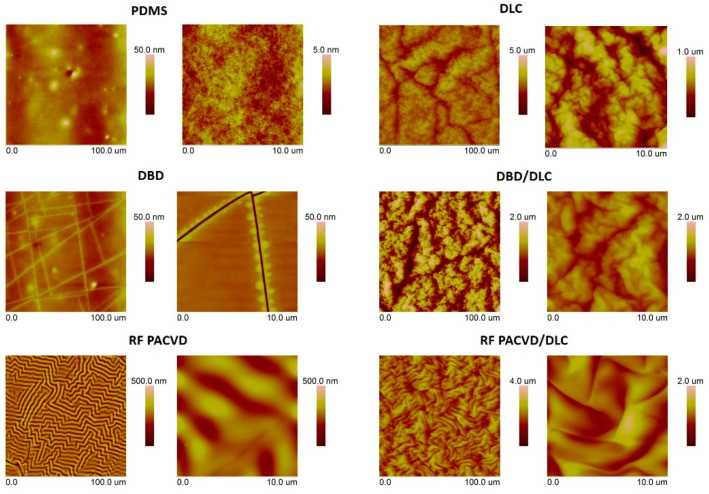
On the left, the AFM pictures show the surface of the polydimethylsiloxane (PDMS) substrate: unmodified, subjected to 60 s modification in DBD air plasma, and subjected to 60 s modification in low-pressure radio-frequency plasma-assisted chemical vapour deposition (RF PACVD) plasma. On the right, the pictures show the same substrates coated with a DLC layer.

The most noticeable effect of plasma on the PDMS polymer substrate was the changes in the geometric structure of its surface. Figure 1 presents AFM images showing the impact of every type of modification in comparison with an unmodified material.

As shown, various geometric structures, with different surface development, can be produced depending on the type of plasma used for plasma pre-treatment. The observed changes in the geometry of the modified substrate surfaces can be a reflection of the different temperatures of the treatment processes in RF and DBD plasma. Modifications involving DBD plasma are low-temperature processes [26,27,40], which do not cause significant changes in the geometric structure of the modified PDMS. In the case of modifications performed using RF PACVD equipment, the temperature of the modified surface can exceed 100 °C [42]. This is also confirmed by the measured roughness parameters shown in Table 2. After an initial 60 s modification in DBD plasma, there were many lines visible on the PDMS surface, resulting from cracks in the oxide layer produced at this stage. This process has a minor impact on the roughness parameters (except for the Rz parameter, which is closely associated with cracks on the surface), which may indicate the polymer-like nature of the coating. In the case of the initial 60 s treatment in radio-frequency plasma, there were noticeable wrinkles on the modified surface, resulting in a 40-fold increase in surface roughness parameters in comparison with the unmodified specimen. It can be assumed, therefore, that the oxide coating produced under such conditions has a different Young’s modulus from the polymer material. The observed effect is closely associated with the effect of RF plasma on the PDMS surface, which is why similar changes can be observed in the geometric structure of the surface after the production of the DLC coatings. The impact of the initial modification on the changes in the surfaces modified with DLC coatings can be observed in the AFM pictures. The pictures show that the less developed the surface, the finer the cauliflower structure. The surface of the DLC coatings produced on the untreated PDMS surface (with the lowest roughness parameters) is characterised by the most wrinkled geometry for the microscopic regions (the estimated wavelength of the wrinkles λ was equal to 2.3 µm, and their amplitude A was 76 nm). In the case of the DLC coatings produced on the PDMS substrate subjected to pre-treatment in radio-frequency plasma (with the highest roughness parameters), the wrinkles created on the surface were described by λ = 4.8 µm and A = 220 nm. The parameters obtained for the DLC coatings deposited on the substrates treated in DBD plasma were λ = 3.5 µm and A = 140 nm. Additionally, data from Table 2 show that the use of plasma pre-treatment resulted in an increase in roughness parameters by at least 12% in comparison with the specimens with DLC coating produced directly on an unmodified PDMS substrate. The highest roughness parameters could be observed in the specimen with the DLC coating pre-modified in DBD plasma, which was most likely caused by the many cracks on its surface, which intensified plasmo-chemical processes.

### 3.2. Changes in the Water Contact Angle before and after the Production of DLC Coatings

**Figure 2:**
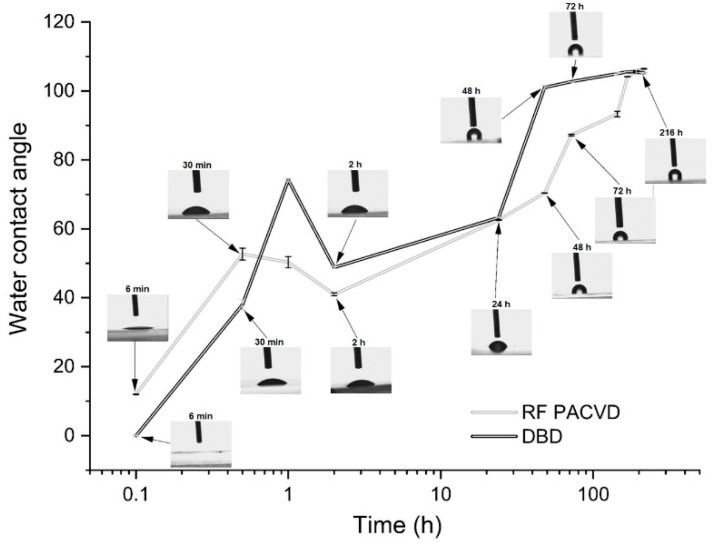
Changes in the water contact angle with time after one-minute plasma treatment in air using the DBD and RF PACVD techniques.

Changes occurring on the surfaces of PDMS substrates modified with plasma can be observed by monitoring the water contact angle. Figure 2 shows the changes in the water contact angle with time after 60 s plasma treatment using the RF PACVD and DBD techniques. They are indicative of physical and chemical processes taking place on the polymer surface long after the process is over. Stabilisation is not observed until the lapse of 3 days.

**Figure 3:**
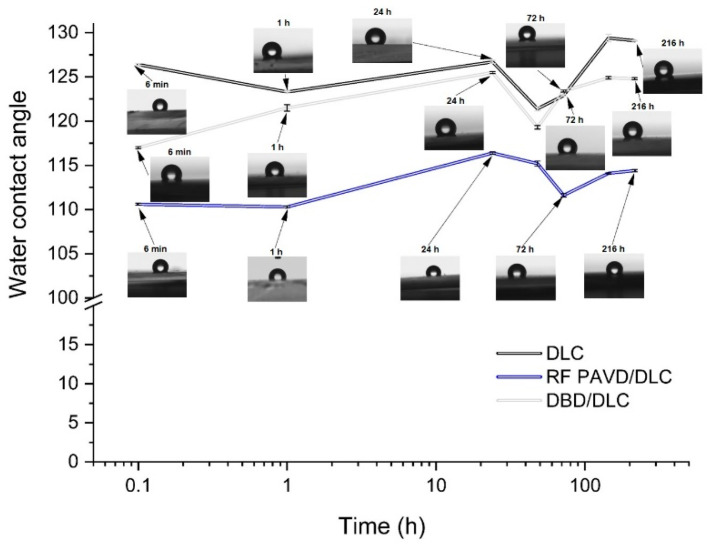
Changes in the water contact angle for substrates with the DLC coating.

It should be emphasised that the unmodified PDMS substrate has a water contact angle in the range of 115.6° ± 0.23°. As shown in the figure, directly after the plasma treatment processes, the water drop spread onto the surface or reached an angle of several degrees. Over time, the angle increased (at a similar rate for both processes) and reached, after several days, a value of 105°. The changes in the water contact angles for the substrates with the DLC coating were different (Figure 3). Throughout the tests, the angles oscillated around a constant level characteristic for the produced geometric structure of the surface. For the DLC coatings produced directly on the PDMS substrate, the average water contact angle was 125.6°; when substrates subjected to RF PACVD plasma pre-treatment were used, the angle was 113.2°, and for substrates subjected to DBD plasma pre-treatment, 122.3°.

### 3.3. Chemical Structure Analysis

**Figure 4:**
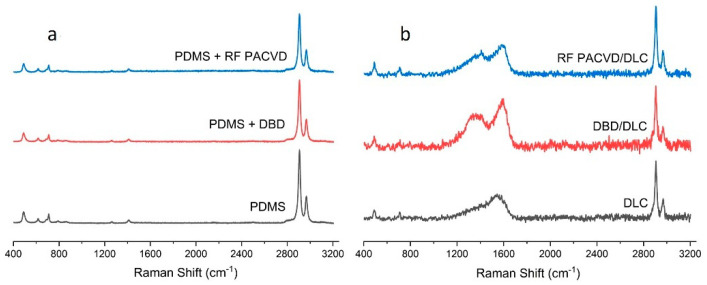
Raman spectra characteristic of (**a**) unmodified polymer substrate and also substrate after 60 s pre-treatment using DBD or RF PACVD plasma, and (**b**) after the production of DLC coatings.

The chemical structure of the PDMS substrates before and after modification was tested using Raman spectroscopy. This technique can determine the characteristics of both the polymer substrate and the DLC coatings produced on the substrate. Figure 4a shows the Raman spectra for the unmodified polymer substrate and for a substrate subject to 60 s pre-treatment in RF PACVD and DBD plasma.

As shown, the presented spectra did not differ from each other. The analysis identified the following peaks in the following positions: 488 cm^−1^ associated with Si-O-Si symmetric stretching, 618 cm^−1^ associated with Si-C symmetric stretching, 687 cm^−1^ associated with Si-CH_3_ symmetric rocking, 708 cm^−1^ associated with Si-C symmetric stretching, 1262 cm^−1^ associated with CH_3_ symmetric bending, 1412 cm^−1^ associated with CH_3_ asymmetric bending, 2907 cm^−1^ associated with CH_3_ symmetric stretching and 2965 cm^−1^ associated with CH_3_ asymmetric stretching [43,44]. Figure 4b shows the spectra produced after the deposition of the DLC coatings, recorded in the same measuring range as for the substrates before the modification.

**Figure 5:**
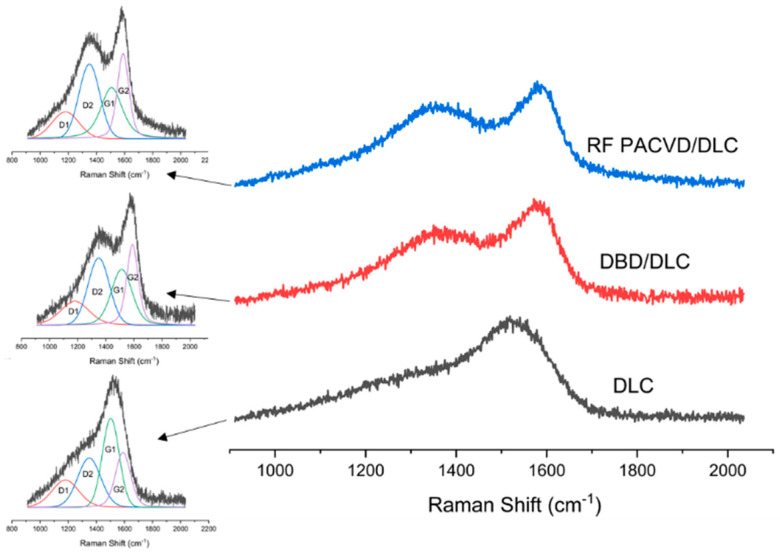
Raman spectra for DLC layers, including their deconvolution into four characteristic peaks.

Figure 5 shows the spectra of the DLC coatings in the range of 1000 to 2000 cm^−1^, including their deconvolution into four characteristic peaks [17,45]. The conducted analyses were used to determine the ID/IG ratios for the produced DLC layers. The smallest ID/IG ratio, i.e., 0.80, calculated as the quotient (ID1 + ID2)/(IG1 + IG2), was recorded for the DLC coating produced directly on the PDMS substrate. The ID/IG values for the remaining coatings amounted to 0.91 for the specimen with pre-treatment in RF PACVD plasma and 0.89 for the specimen with pre-treatment in DBD plasma. A thorough analysis of the spectra generated by the DLC coatings, deconvolutions and calculated ID/IG ratios indicates that the DLC created directly on the PDMS substrate had the lowest ID/IG ratio, which is indicative of the highest content of C-C sp3 bonds according to relevant studies [46,47,48]. The DLC coating produced on the substrates subjected to plasma pre-treatment, in turn, had a similar higher ID/IG ratio, i.e., a lower content of sp^3^ bonds typical of a diamond-like structure. According to the work of Casiraghi [49], the hydrogen content in the coatings can be estimated to be around 13%. Additionally, based on our previous publication [38], it can also be determined that that the sp^3^ content in the produced DLC coatings is expected to be below 30%.

### 3.4. Analysis of the Coefficient of Friction

**Figure 6:**
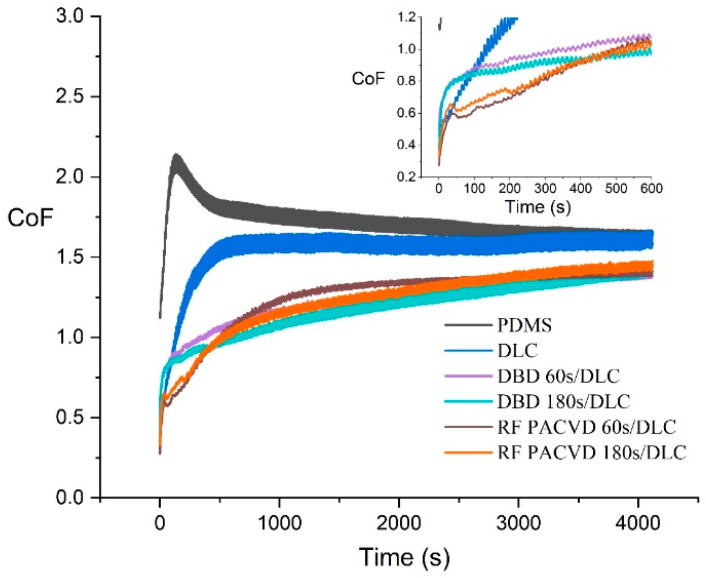
Changes in the coefficient of friction of substrates used in the tribological tests.

Tribological tests were carried out for PDMS substrates modified with DLC coatings using different pre-treatment processes and, for comparison, for substrates without pre-treatment. To determine the impact of the duration of plasma pre-treatment, the tests included substrates subject to 60 and 180 s treatments. Figure 6 shows the changes in the coefficient of friction over time for all the analysed specimens. As shown, in comparison with pure PDMS, all the modifications had a positive effect on the reduction of the coefficient of friction. The highest impact on the reduction was recorded when plasma pre-treatment in the DBD or RF PACVD system had been used. In the case of the unmodified PDMS substrate, the adopted conditions for the tribological tests resulted in an initial increase in CoF to 2.1, followed by its stabilisation at 1.6. The tests of the specimen with the DLC coating produced without pre-treatment showed small initial values of CoF (approximately 0.2), but, unfortunately, the parameter increased to 1.6 only 500 s after the beginning of the test. As shown by our previous results [16], such a course of CoF change points to a low adhesion of the produced coating to the substrate. In a short period of time, catastrophic wear occurs, resulting in a sudden increase in the CoF value. The changes in the coefficient of friction were different for the PDMS substrates with DLC coatings produced on surfaces subjected to pre-treatment. These specimens showed a slow increase in the coefficient of friction during the test cycle to a level of 1.4. The charts are different depending on the type of plasma modification used (RF PACVD or DBD), particularly during the initial stages of the tests, before the lapse of 400 s. Figure 6 shows that the coefficient of friction of the specimen with the DLC coating subjected to an additional 60 s pre-treatment in DBD plasma was the lowest in the first phase. The results are only slightly worse for the specimen modified in the same plasma for 180 s. The changes in the coefficient of friction in the time range of 0–300 s for the specimens pre-treated in radio-frequency plasma were initially higher, but, over time, they reached levels similar to those for the specimens modified in DBD plasma.

### 3.5. Wear Rate

**Figure 7:**
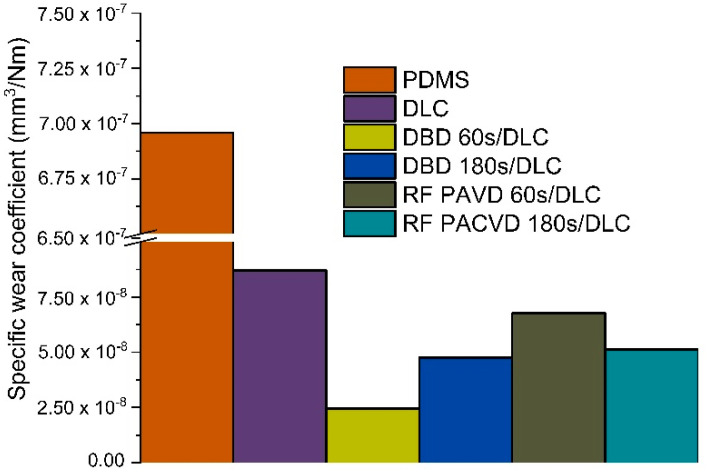
Analysis of wear rates achieved for substrates used in the tribological tests.

Figure 7 shows the calculated wear rates, in accordance with methodology described in our previous publications [16,17]. It can be observed that the applied modifications of the PDMS material significantly reduced this parameter. The wear rate for an unmodified PDMS specimen is 6.96 × 10^−7^ mm^3^/Nm. The DLC coatings produced without initial etching showed a wear rate of 8.70 × 10^−8^ mm^3^/Nm. The lowest value after the modification—2.45 × 10^−8^ mm^3^/Nm—was achieved for the PDMS specimen with the DLC coating pre-etched for 60 s in barrier plasma. The wear rate for the specimen etched with the same type of plasma, but for 180 s, was 4.76 × 10^−8^ mm^3^/Nm. The wear rates for the specimens pre-treated in RF plasma were higher than after treatment in barrier plasma, reaching a value of 6.77 × 10^−8^ mm^3^/Nm after 60 s of etching and 5.12 × 10^−8^ mm^3^/Nm after 180 s.

### 3.6. Wear Analysis

**Figure 8:**
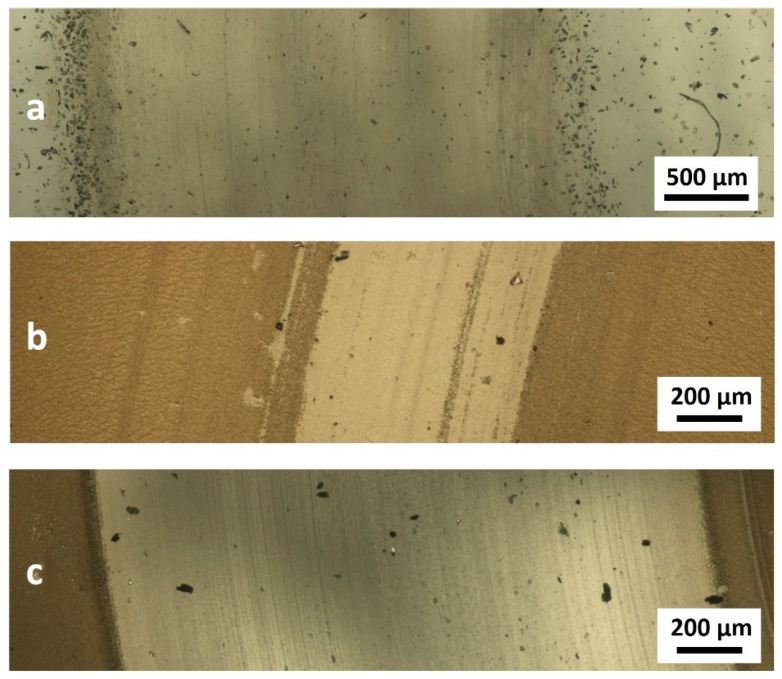
Sample forms of wear of the specimens after the tribological tests (**a**) for an unmodified PDMS specimen, (**b**) for a specimen modified through the RF PACVD 60 s/DLC process and (**c**) for a specimen modified with the DLC coating.

After the tribological tests, the specimens were subject to surface tests, where the wear marks were assessed using optical microscopy. The friction between the ceramic ball and the unmodified PDMS substrate produced a large amount of wear products, pushed outside the wear area (Figure 8a). Our earlier tests on the application of DLC coatings for modifying polymer substrates showed that their wear rates were variable over time [17]. In the initial phase, the coating was being smoothed, and the worn-out fragments filled the irregularities in the surface. Subsequent processes contributed to the bonding of the wear products and formation of larger particles, which may have caused discontinuity in the coating. In the presented publication, regardless of the applied parameters of plasma treatment, removal of the coating with wear marks was observed after the tribological processes, as shown by Figure 8b,c. The pre-treatment of the PDMS substrates resulted in limited wear. In these cases, partial wear of the DLC coating was observed (Figure 8b), while the DLC coatings deposited on the untreated polymer substrate were fully removed within the wear area.

## 4. Discussion

The production of DLC coatings with good tribological properties on polymer substrates requires an initial modification of the surfaces of such materials, particularly in the case of soft materials such as PDMS. These processes may be conducted using both radio-frequency and DBD plasma. The study shows that each of the indicated techniques has a different effect on the development of the surface of PDMS substrates (Figure 1). After the DLC coatings are produced on the modified substrates, the geometric parameters of the produced surfaces are similar (Table 2). However, as shown by the AFM pictures in the 10 × 10 µm areas, the least developed surface was produced for DLC coatings deposited on an unmodified PDMS substrate; the most developed surface was produced on the specimen with the DLC coating created after etching in RF plasma. The differences affect the water contact angles of the tested surfaces. The most developed surface had the smallest water contact angle (Figure 3). The tests showed that the effects of the plasma pre-treatment of PDMS substrates are unstable over time. However, after coating the modified substrates with the DLC coating, the changes can be halted, as confirmed by the stability of the water contact angle over time. An analysis of the chemical structure of the modified PDMS substrates shows that the applied plasma pre-treatment processes did not cause any major changes in the chemical structure. The Raman spectra presented in Figure 4 on the left show typical peaks characteristic of the chemical structure of PDMS [43,44]. However, after the production of the DLC coatings, there were noticeable differences between the coating produced directly on the PDMS substrate and the coatings produced on a substrate subjected to plasma pre-treatment. As shown in Figure 6, carbon coatings with a higher ID/IG ratio (lower content of the sp^3^ phase) had smaller coefficients of friction throughout the test cycle. The best of the compared modifications was the specimen subjected to treatment using DBD plasma for 60 s. When combined with the production of the DLC carbon coating, it allowed for the achievement of a wear rate of 2.45 × 10^−8^ mm^3^/Nm. However, the effects achieved using RF plasma do not disqualify this technology. The changes in the coefficient of friction indicate that plasma pre-treatment processes have a positive impact. They can be used to improve the tribological properties of the modified surfaces. This is closely related to the phenomena occurring during such processes. Plasma pre-treatment causes surface cross-linking, resulting in a superficial increase in surface hardness. This effect significantly improves the functional properties of the DLC coatings created during the next stage [16]. Although the research in this paper shows that the best solution is to use barrier plasma, the applications of this technology seem limited due to difficulties with the cooling of the specimens in these processes. The further extension of the duration of the pre-treatment of PDMS substrates in DBD plasma may lead to their degradation. Unlike the DBD technology, RF PACVD systems use a cooled high-frequency electrode, which enables much more extensive control of plasma pre-treatment processes. Another important advantage of pre-treatment in RF plasma is the fact that both processes, i.e., pre-treatment and the production of the DLC coating, can be performed in a single chamber without removing the substrates.

Examinations of the surface conducted after the tribological tests showed that the carbon coatings produced on the PDMS surfaces, whether modified or not, suffered from damage. However, it should be emphasised that the testing conditions were harsh for materials such as PDMS. Despite the damage process, the coefficient of friction of the substrates pre-treated in RF and DBD plasma did not increase to the levels recorded for unmodified PDMS. This confirms the results of our earlier research and shows that the process significantly depends both on the DLC coating and on the layer produced directly underneath, during plasma pre-treatment. As shown by our earlier experiences, the production of the described layer is the result of the cross-linking of PDMS substrates [16]. It is precisely because of the layer of cross-linked polymer under the DLC coating that the specimens of modified PDMS we tested (referred to as RF PACVD/DLC and DBD/DLC) have better tribological properties and wear resistance than the other specimens (Figure 7 and Figure 8).

## 5. Conclusions

The plasma pre-treatment of PDMS substrates has a significant impact on the tribological properties of the DLC coatings produced on the substrates. It can be performed using both radio-frequency plasma and DBD plasma, and each technique produces a different geometric structure of the surface. Carbon coatings produced on substrates subjected to plasma pre-treatment and on substrates that were not treated this way had different contents of C-C sp^3^ bonds. The results provide evidence of the formation of a cross-linked intermediate layer between the DLC coating and the PDMS substrate, which is one of the factors determining the tribological properties and wear resistance.

## Figures and Tables

**Table 1:** Parameters of modification processes.

Series Number	Preliminary Plasma Modification				Deposition of DLC Coatings	
	*RF Plasma*		*DBD Plasma*			
	Time (s)	Type of Atmosphere	Time (min)	Type of Atmosphere	Time (s)	Type of Atmosphere
**1**					300	CH_4_
**2**	60	Air			300	CH_4_
**3**	180	Air			300	CH_4_
**4**			60	Air	300	CH_4_
**5**			180	Air	300	CH_4_

**Table 2:** Roughness parameters of the surface of the PDMS substrate: unmodified, and modified in RF PACVD plasma and in DBD plasma, and, furthermore, the same surfaces with DLC coatings.

Sample	Surface Parameters		
	Ra (nm)	Rz (nm)	Rq (nm)
**PDMS**	1.09 ± 0.604	6.73 ± 5.65	1.48 ± 1.02
**DBD**	1.59 ± 0.566	26.8 ± 8.84	3.09 ± 1.16
**RF PACVD**	43.4 ± 7.76	225 ± 25.3	52.4 ± 7.91
**DLC**	186 ± 35.3	965 ± 169	229 ± 41.1
**DBD/DLC**	256 ± 39.6	1330 ± 192	315 ± 48.1
**RF PACVD/DLC**	220 ± 30.5	1082 ± 137	265 ± 34.7

The grey colour is used to mark roughness parameters after initial plasma modifications.

## Data Availability

The data used to support the findings of this study are available from the corresponding author upon request.
